# Research on the Microstructures and Mechanical Properties of Bainite/Martensite Rail Treated by the Controlled-Cooling Process

**DOI:** 10.3390/ma12193061

**Published:** 2019-09-20

**Authors:** Jiajia Qiu, Min Zhang, Zhunli Tan, Guhui Gao, Bingzhe Bai

**Affiliations:** 1 School of Mechanical, Electronic and Control Engineering, Materials Science and Engineering Research Center, Beijing Jiaotong University, Beijing 100044, China; 17121353@bjtu.edu.cn (J.Q.); tzli@bjtu.edu.cn (Z.T.); gaogh@bjtu.edu.cn (G.G.); bzbai@bjtu.edu.cn (B.B.); 2 Key Laboratory of Advanced Materials of Ministry of Education, School of Materials Science and Engineering, Tsinghua University, Beijing 100084, China

**Keywords:** bainite/martensite multiphase rail, high-angle boundary, controlled-cooling process, decomposed martensite, crack initiation

## Abstract

A bainite/martensite multiphase rail is treated by the controlled-cooling process with different finish-cooling temperatures. The simulated temperature–time curves of the position of 5 mm and 15 mm below the rail tread (P5 and P15) express different trends. P5 has greater impact toughness and lower tensile strength than P15. Microstructural characterization was carried out by conducting scanning electron microscopy, X-ray diffraction, electron backscatter diffraction, and transmission electron microscopy. The greater tensile strength is due to the dispersed ε-carbides hindering the movement of dislocations. The greater impact toughness is attributed to the filmy retained austenite and the smaller effective grain with high-angle boundary. Finite element modeling (FEM) and microstructural characterization reasonably explain the changes of mechanical properties. The present work provides experimental and theoretical guidance for the development of rail with excellent mechanical properties.

## 1. Introduction 

Heavy-duty and high-speed railway transportation brings about increased stress on rail and worsening operating conditions. Rail damage is also increased, such as peeling, concealing or even fracture, due to wear and fatigue. Therefore, it is important to increase the strength of rail that can improve wear resistance and prolong service life. The research and development of next-generation rail focuses on enhancing mechanical properties [1,2]. Low-carbon bainite/martensite (B/M) multiphase rail has been successfully used in some heavy haul railway and has much potential to substitute the traditional pearlite rail due to its excellent mechanical properties [3,4,5]. The conventional production process of B/M multiphase rail is air-cooling after hot rolling, which is susceptible to seasonal and regional impact. The controlled-cooling process can reduce the impact of environment and improve the uniformity of microstructures. It has been proved to refine the intragranular microstructure of steel, which in turn improves mechanical properties, such as strength and impact toughness [6,7]. 

In recent years, finite element modeling has been widely applied in the manufacturing of rail. It can simulate the microstructure evolution and temperature changes in the different positions of rail, which contributes to design and optimization of the cooling process after hot rolling [8,9]. Changing the cooling process could yield different types of microstructural phases (e.g. bainite ferrite, martensite, retained austenite, and even ε-carbide) coexisting as microstructural constituents in B/M multiphase rail steel, which influence mechanical properties through its morphology, content, and size [10]. A number of studies have shown that the effective grain with high-angle boundary can disrupt crack propagation, i.e., the propagation direction changes at these boundaries and the crack propagation path increases [11,12,13]. Filmy retained austenite (RA) with higher carbon content has superior stability and acts as an obstacle during crack propagating [14,15,16]. That can be favorable to improve the service life of rail steel. A large number of dispersed ε-carbides can hinder the movement of dislocations and produce precipitation strengthening, which are good for the improvement of mechanical properties [17,18]. 

The present study focuses on the microstructures and mechanical properties of the industrially produced B/M rail steel through the controlled-cooling process with different finish-cooling temperatures. Due to microstructure segregation and the largest shear stress, the actual engineering application pays more attention to the microstructure and mechanical properties of the position of 5 mm and 15 mm below rail tread. The effect of microstructures on mechanical properties in these positions has been researched in-depth, involving electron microscopy, tensile, and impact test. The work can provide a scientific and systematic guidance for the development of the high strength and toughness rail.

## 2. Experimental Procedures

The chemical composition of bainitic/martensite rail steel is 0.22C–2.0Mn–1.0Si–0.8Cr–0.8 (Mo + Ni) (wt%). The spray cooling process was adopted to control cooling speed of the rail-head after rolling. The surface of the rail-head was cooled by the spray mist, while the rest of the rail was naturally cooled. When the surface temperature of the rail-head decreased to finish cooling temperatures (Tq) of 230 °C, 250 °C, and 280 °C (abbreviated T230, T250, and T280, respectively), the spray controlled-cooling process was stopped, and then the whole rail adopted the air-cooling process. 

The volume fraction of RA (X_RA_) was measured by X-ray diffractometer (Rigaku Smartlab, Rigaku Corporation, Tokyo, Japan, Cu Ka radiation) at a step of 0.01° and a counting time of 2 s/step using 10 mm × 10 mm × 2 mm samples. X_RA_ was estimated by collecting the peak intensities of (200)γ, (220)γ, (311)γ, (200)α, and (211)α. The precise austenite lattice constant $\left(a_{\gamma }\right)$ was obtained by Nelson-Riley extrapolation method. The carbon content of retained austenite (X_C_) can be estimated using Equation (1):

(1) $$a_{\gamma }=3.556+0.0453\;x_{C}+0.00095\;x_{\mathrm{Mn}}+0.0056\;x_{\mathrm{Al}}$$

where the austenite lattice $a_{\gamma }$ is in Å, and $x_{C}$, $x_{\mathrm{Mn}}$, and $x_{\mathrm{Al}}$ are the concentrations of carbon, manganese, and aluminum, respectively, in wt %.

Microstructures were characterized by scanning electron microscopy (SEM; ZEISS-EVO18, 20 kV, Carl Zeiss Microscopy Ltd., Cambridge, UK) after polishing and etching in 3% nital solution. The specimens for transmission electron microscope (TEM; JEOL F200, 200 kV, JEOL, Tokyo, Japan) were electropolished at −30 °C using 6% perchloric acid solution. Auger electron spectroscopy (AES, ULVAC-PHI, Inc., Chigasaki, Japan) was applied for electron backscatter diffraction (EBSD) measurements utilizing a PHI 710 microprobe operating (ULVAC-PHI, Inc., Chigasaki, Japan) at 20 kV with a step size of 0.1 μm. And the scanning area is 40 μm × 40 μm, which can cover about five prior austenite grains.

**Figure 1:**
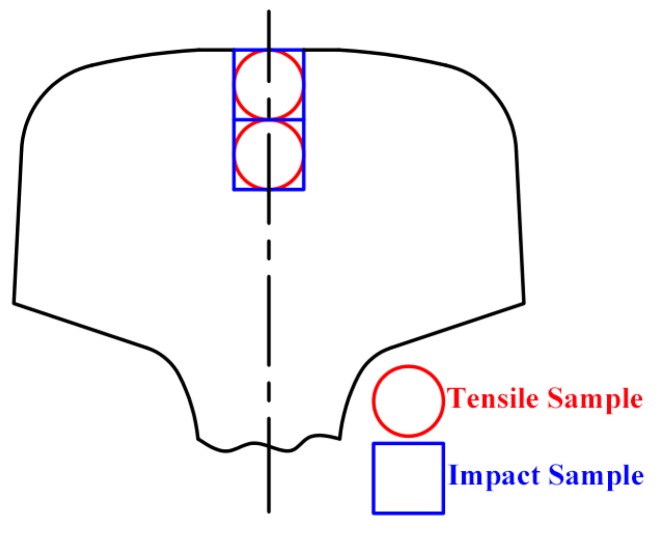
Schematic diagram showing the sampling positions in the rail-head.

Specimens were cut from the rail-head in the rolling direction as shown in Figure 1. Standard tensile samples with a gage diameter of 5 mm and a gage length of 25 mm were prepared for tensile tests using a SUNS 5305 tensile testing machine (MTS Systems, MTS, Shanghai, China). Two samples were tested for each process. An extensometer and a force sensor were used. Impact tests were performed using standard Charpy U-notch specimens (10 mm × 10 mm × 55 mm, standard EN10045) using JBDS-300B impact tester device (Wuzhong Material Test Ltd, Wuzhong, China) at 20 °C. Three specimens were used for each test. 

## 3. Results and discussion

### 3.1. FEM Simulation of the Controlled-Cooling Process

**Figure 2:**
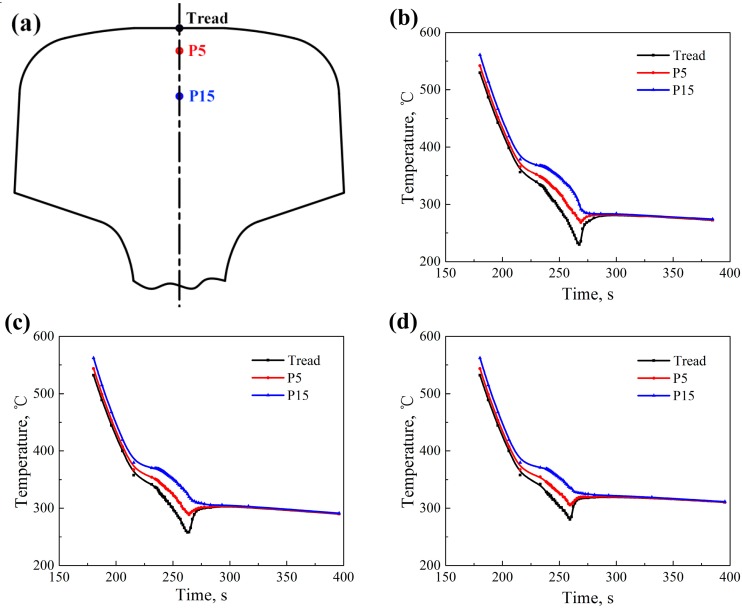
Finite element modeling (FEM) simulation results: (**a**) The sampling positions in the rail-head (Tread, P5, and P15); (**b**–**d**) Temperatures with the cooling time in different processes (T230, T250, and T280, respectively).

The controlled-cooling processes of T230, T250, and T280 after rolling are simulated by finite element modeling (FEM). Temperatures of different positions such as tread, the position of 5 mm below rail tread (P5), and the position of 15 mm below rail tread (P15), as indicated in Figure 2a, can be obtained. Part of temperatures with respect to the cooling time in the controlled cooling processes (T230, T250, and T280) are respectively presented in Figure 2b–d. The cooling rate at each position of rail-head reduce due to the latent heat of phase change when the temperature decreases below 400 °C. The temperature of tread is about 50 °C lower than that of P15. The curves of temperature–time of different positions in rail-head, shown in Figure 2b–d, express different trends when the surface of rail-head is changed from the spray controlled-cooling process to the air-cooling process. Temperatures of tread and P5 begin to increase due to the latent heat of phase change and the internal heat transfer while temperatures of P15 decrease very slowly. Then temperatures at all positions reach a stable plateau, which lasts for about 10 min.

### 3.2. Mechanical Properties

The mechanical properties at different tread-head positions treated by different processes are summarized in Table 1. All rail samples exhibit a good combination of tensile strength (above 1320 MPa) and impact toughness (above 90 J). The impact toughness of P5 is about 30 J greater than that of P15. Furthermore, the T280-P5 sample reaches a maximum value of 131 J. The tensile strength of P5 decreases from 1402 MPa to 1322 MPa and that of P15 from 1504 MPa to 1379 MPa when the Tq increases from 230 °C to 280 °C. At the same time, the yield strength of P5 decreases from 1295 MPa to 1201 MPa and that of P15 from 1272 MPa to 1181 MPa. The strength of samples increases as the Tq decreases, which is caused primarily by the different martensite content in the samples. The content of the prior martensite increases with the decrease of finish cooling temperature as Koistinen-Marburger relationship describes. Usually the larger martensite amount results in a higher strength. The tensile strength of P5 is about 50 MPa lower than that of P15 in the same rail. For samples of P5, the residual internal stress may get decreased and carbon atoms diffuse out of the martensite when temperature increases after the spray controlled-cooling process completed, which may reduce the tensile strength of the samples [19]. 

### 3.3. Microstructure Characterization

**Figure 3:**
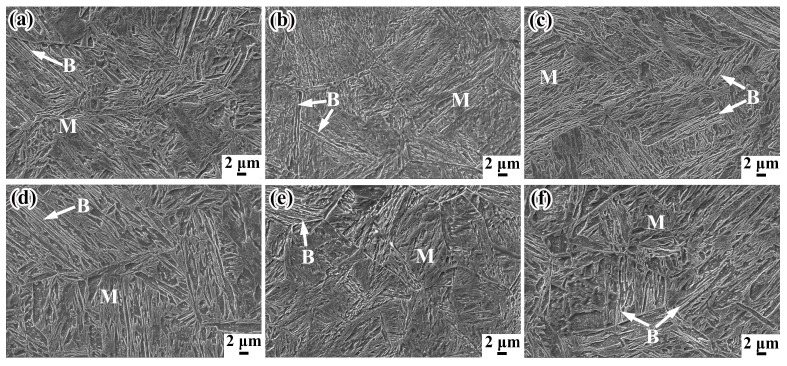
Scanning electron microscopy (SEM) of samples at the positions of 5 mm and 15 mm below rail tread treated by different process: (**a**) T230-P5; (**b**) T250-P5; (**c**) T280-P5; (**d**) T230-P15; (**e**) T250-P15; (**f**) T280-P15.

The SEM micrographs of samples at the positions of 5 mm and 15 mm below rail tread, taken from the rolling direction of rails, are respectively shown in Figure 3. The microstructures at different positions contain martensite and a small amount of bainite and retained austenite. The lath-like microstructures of samples are clearer and significantly finer with the finish-cooling temperature decreasing. The lower the finish-cooling temperature, the larger the nuclear driving force of phase transformation. The probability of mutual interference and collision between the growing laths increases, reducing the size of the lath martensite [20], which results in greater strength of T230 samples compared with that of T280 samples. According to the continuous cooling transformation (CCT) curves of rail steel shown in reference [21], changing cooling rates could yield different types of microstructures. The content of martensite increases with the increasing of cooling rate in the continuous-cooling process. The martensite content of P5 is more than that of P15 because the cooling rate close to the surface of the rail-head is faster. Interestingly, more martensite content does not result in greater tensile strength. 

**Figure 4:**
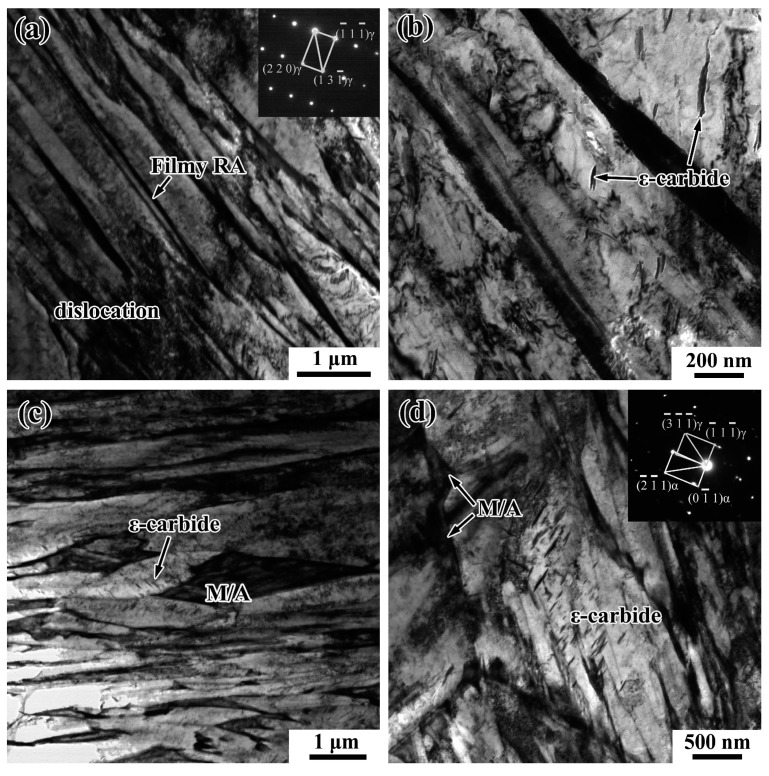
Transmission electron microscope (TEM) of samples at the positions of 5 mm and 15 mm below rail tread treated by T280 process: (**a**,**b**) T280-P5; (**c**,**d**) T280-P15.

Figure 4 indicates the TEM micrographs of samples at the positions of 5 mm and 15 mm below rail tread treated by the T280 process. Figure 4a shows that the morphology of retained austenite in the T280-P5 sample is almost filmy and it distributes between martensite laths. Figure 4d expresses that the retained austenite of the T280-P15 sample exists in blocky martensite/austenite (M/A) constituents distributing around martensite laths. The size of blocky M/A constituents is much larger than that of filmy RA. The filmy RA is more mechanically stable than the blocky ones because of the higher carbon content. The blocky M/A constituents are easily transformed into martensite at low temperature or by exertion with external force, which may become crack sources resulting in lower impact toughness. It has been reported that the filmy RA can improve the impact toughness by inhibiting the formation of micro-cracks and suppressing the crack propagation [10,22]. There is a small amount of ε-carbide precipitation within the ferrite plates and they form parallel arrays at about 60° to the axis of the ferrite plates, as shown in references [23,24]. Figure 4c,d indicate that the ε-carbide precipitation in the T280-P15 sample is much more than that in the T280-P5 sample. A large number of dispersed ε-carbides can hinder the movement of dislocations and produce precipitation strengthening, which may explain why the tensile strength of P15 is greater than that of P5.

**Figure 5:**
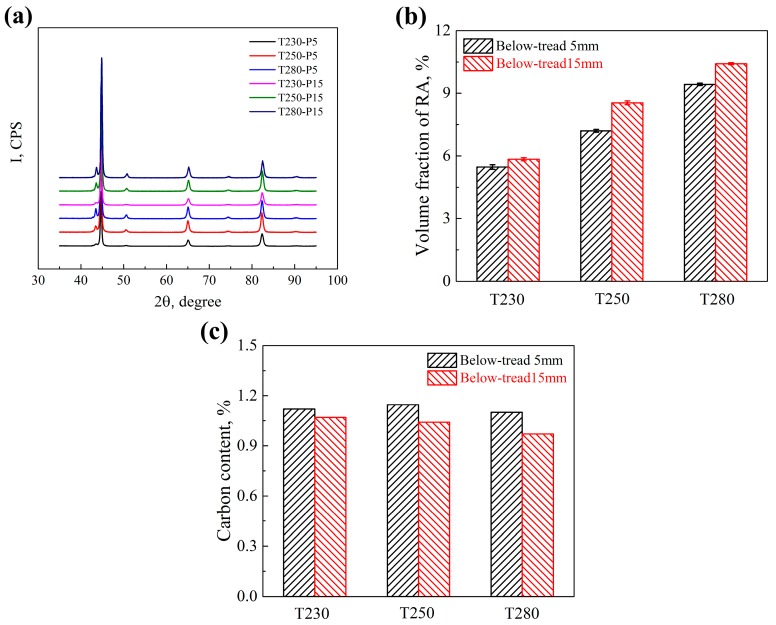
XRD analysis results: (**a**) XRD spectra; (**b**)Volume fraction of retained austenite; (**c**) Carbon content of retained austenite.

XRD patterns of different samples are shown in Figure 5a. The volume fraction of retained austenite and carbon content of retained austenite are presented in Figure 5b,c. X_RA_ increases with the Tq increasing from 230 °C to 280 °C. The samples of P15 have greater X_RA_ than samples of P5 in the same rail. The smaller Tq, the greater content of austenite transforming to martensite, which leads to smaller volume fraction of retained austenite. Carbon atoms will diffuse from martensite with higher chemical potential to retained austenite when the spray controlled-cooling process changes to the air-cooling process, which can increase the carbon content of retained austenite. The retained austenite with lower carbon content will continue to undergo phase transformation to form martensite, while the one with sufficient carbon content will become more stable. The X_C_ of the P5 samples is greater than that of the P15 samples in the same rail. As discussed earlier, the samples of P5 have much more ε-carbide precipitation than the samples of P15. The carbide precipitation increases the carbon consumption, which results in greater X_C_ of the P5 samples. 

### 3.4. Effect of the Microstructures on the Impact Toughness

**Figure 6:**
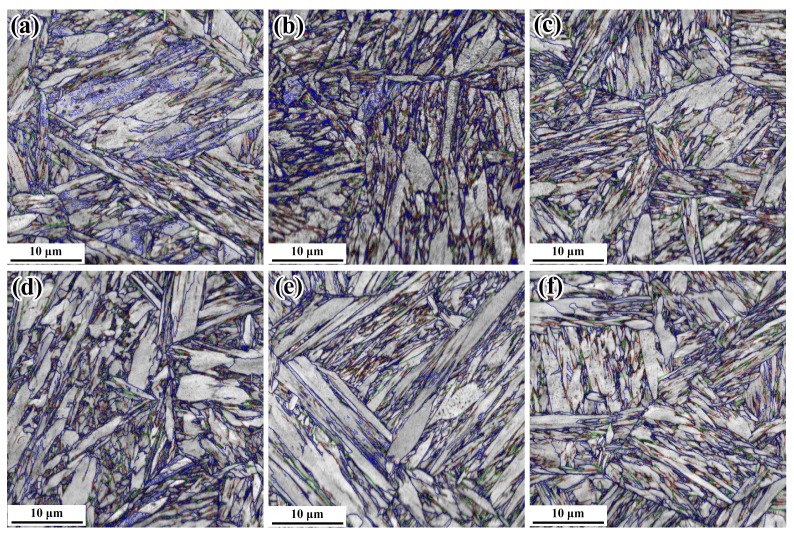
The grain boundaries of samples at the positions of 5 mm and 15 mm below rail tread treated by different process: (**a**) T230-P5; (**b**) T250-P5; (**c**) T280-P5; (**d**) T230-P15; (**e**) T250-P15; (**f**) T280-P15.

EBSD results are presented in 6,7 to discuss the effect of microstructures on the impact toughness. Figure 6 indicates the grain boundaries (red lines: 2–5°, green lines: 5–15°, blue lines: >15°) of samples at the positions of 5 mm and 15 mm below rail tread treated by different controlled-cooling processes. 

**Figure 7:**
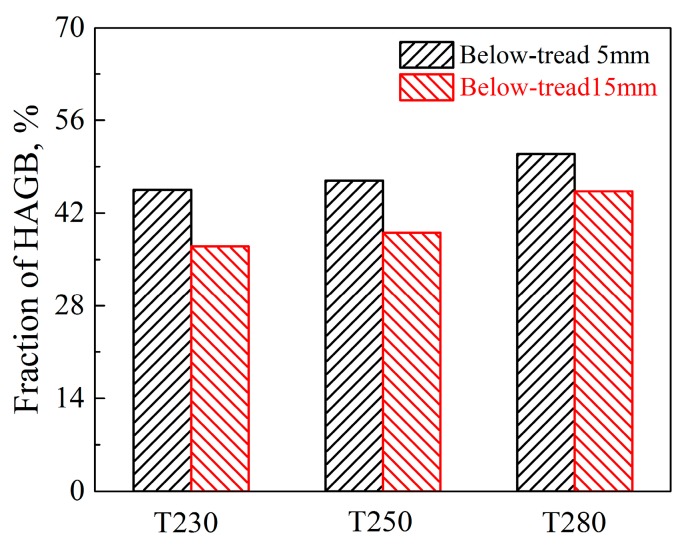
Fraction of high-angle (>15°) grain boundaries (HAGBs).

As shown in Figure 7, the samples of P5 have a higher proportion of high-angle grain boundaries (misorientation >15°) than the samples of P15. The grain with high-angle grain boundaries (HAGBs) is described as effective grain, as they play an important role in blocking crack propagation [21]. In lath martensite, the prior austenite grain is divided into several martensite packets with the same habit plane, and each martensite packet is divided into several martensite blocks with close orientation. The boundaries of packet and block are both high-angle grain boundaries [25,26]. The smaller the size of packet and block per unit area, the higher the fraction of the high-angle grain boundaries. The effective grain size of the P5 samples is smaller than that of the P15 samples due to the faster cooling-rate, which may improve the fraction of HAGBs [27]. The further transformation of retained austenite after the spray controlled-cooling process makes some filmy RA transform into filmy M/A constituents with more HAGBs. High-angle grain boundaries have much more grain boundary energy than that of low-angle grain boundaries because a greater number of atoms deviate from its equilibrium position. The irregular arrangement of the atoms may cause the directions of crack propagation to change multiple times and much more crack propagation energy to be consumed [28]. Therefore, HAGBs can be more effective to force the crack to change the propagation directions, hinder the cracks propagation and improve impact toughness. As such, the impact toughness of the low-carbon B/M multiphase rail steel is greatly affected by the fraction of HAGBs.

## 4. Conclusions

In the present work, the relationship between the microstructures and mechanical properties of the B/M multiphase rail treated by the controlled-cooling process has been investigated and analyzed. The controlled-cooling processes after rolling was simulated by finite element modeling. The main conclusions can be summarized as follows:

(1) The samples of P5 exhibit greater toughness than the samples of P15 due to the filmy retained austenite with higher carbon content and the smaller effective grain with high-angle boundary.
(2) The B/M multiphase rails treated by the controlled-cooling process with different finish-cooling temperatures exhibit a good combination of tensile strength (above 1320 MPa) and impact toughness (above 90 J). This work provides scientific and systematic guidance for the development of high strength and toughness rail.

## Figures and Tables

**Table 1:** Mechanical properties of different rail-head positions with different processes.

Condition	Tensile Strength (MPa)	Yield Strength (MPa)	Elongation (%)	Toughness (J)
T230-P5	1402 ± 11	1295 ± 5	13.3 ± 0.1	121 ± 2
T250-P5	1391 ± 2	1243 ± 12	14.5 ± 0.1	124 ± 2
T280-P5	1322 ± 6	1201 ± 6	15.5 ± 0.1	131 ± 2
T230-P15	1504 ± 2	1272 ± 0	12.5 ± 0.1	92 ± 1
T250-P15	1445 ± 3	1215 ± 3	13.8 ± 0.2	97 ± 3
T280-P15	1379 ± 4	1181 ± 0	14.9 ± 0.1	105 ± 0
